# Earlier time to aerobic exercise is associated with faster recovery following acute sport concussion

**DOI:** 10.1371/journal.pone.0196062

**Published:** 2018-04-18

**Authors:** David Wyndham Lawrence, Doug Richards, Paul Comper, Michael G. Hutchison

**Affiliations:** 1 Faculty of Kinesiology and Physical Education, University of Toronto, Toronto, Ontario, Canada; 2 Toronto Rehabilitation Institute, University Health Network, Toronto, Ontario, Canada; 3 Dalla Lana School of Public Health, University of Toronto, Toronto, Ontario, Canada; Cleveland Clinic, UNITED STATES

## Abstract

**Objective:**

To determine whether earlier time to initiation of aerobic exercise following acute concussion is associated with time to full return to (1) sport and (2) school or work.

**Methods:**

A retrospective stratified propensity score survival analysis of acute (≤14 days) concussion was used to determine whether time (days) to initiation of aerobic exercise post-concussion was associated with, both, time (days) to full return to (1) sport and (2) school or work.

**Results:**

A total of 253 acute concussions [median (IQR) age, 17.0 (15.0–20.0) years; 148 (58.5%) males] were included in this study. Multivariate Cox regression models identified that earlier time to aerobic exercise was associated with faster return to sport and school/work adjusting for other covariates, including quintile propensity strata. For each successive day in delay to initiation of aerobic exercise, individuals had a less favourable recovery trajectory. Initiating aerobic exercise at 3 and 7 days following injury was associated with a respective 36.5% (HR, 0.63; 95% CI, 0.53–0.76) and 73.2% (HR, 0.27; 95% CI, 0.16–0.45) reduced probability of faster full return to sport compared to within 1 day; and a respective 45.9% (HR, 0.54; 95% CI, 0.44–0.66) and 83.1% (HR, 0.17; 95% CI, 0.10–0.30) reduced probability of faster full return to school/work. Additionally, concussion history, symptom severity, LOC deleteriously influenced concussion recovery.

**Conclusion:**

Earlier initiation of aerobic exercise was associated with faster full return to sport and school or work. This study provides greater insight into the benefits and safety of aerobic exercise within the first week of the injury.

## Introduction

Concussion guidelines uniformly recommend a brief period (24 to 48 hours) of cognitive and physical “rest” acutely, followed by a gradual and progressive return to activity [1]. The evidence supporting acute rest as an intervention is sparse and largely based on consensus statements, early animal studies, and an understanding of the neurometabolic pathophysiology of concussion [2–4]. Complete or strict rest, protracted rest, and rest until symptom resolution has been shown to be ineffective and potentially hazardous following concussion and is no longer recommended [1, 5–7]. Similarly, acute strenuous physical and cognitive exertion can exacerbate symptom burden, increase risk of secondary injury, and should also be discouraged [8]. However, despite graded re-integration of physical activity being fundamental in concussion management, the optimal type of activity, intensity, duration, and time to initiation following acute concussion are not well elucidated [2–6, 8, 9].

Emerging evidence is documenting a protective effect of active rehabilitative interventions, including physical activity, in the management of concussion [4, 10]. Early introduction of aerobic exercise has been shown to be safe and potentially beneficial in reducing symptom burden and the risk of prolonged symptoms following concussion [8, 11, 12]. Several studies have examined the effect of recommending rest after acute concussion [2–7]; however, there is limited evidence investigating the effect of physical activity in the acute period. In a large prospective cohort study, Grool et al. demonstrated that children and adolescents who engaged in physical activity within 7 days of injury, compared to those who did not, had a reduced risk of persistent symptoms at 28 days [11]. Furthermore, treatment of persistent concussion symptoms with aerobic activity and exercise has demonstrated a protective effect in reducing the severity and prevalence of concussion symptoms [13–17]. Moreover, a recent meta-analysis, including all time points of exercise initiation post-concussion, concluded that aerobic exercise can improve concussion symptom burden following concussion [12].

Investigations to date examining physical activity following acute concussion have been limited in their ability to evaluate the effectiveness aerobic exercise within the 7-day period immediately following a concussive injury [11]. Despite the growing evidence supporting physical activity and aerobic exercise following concussion, additional evidence is required to inform the optimal activity type, intensity, duration, and timing of intervention. The objective of this study was to examine the effect of time to initiation of aerobic exercise on the time to recovery following acute concussion.

## Material and methods

A retrospective study design with consecutive sampling was used to collect data on all acute physician diagnosed concussions presenting to an academic sports medicine clinic from October 2016 to December 2017. The academic sports medicine clinic has ten active sports medicine physicians, all of whom provide concussion care in alignment with the most recent Consensus Statement on Concussion in Sport [1].

All concussions were sport-related and were included if they were ‘acute’, defined for the purpose of this study as presenting 14 days or less following the injury. All ages, sport, and skill levels were included. Concussions were excluded if the index or ‘presenting’ concussive injury occurred during the recovery of a previous concussion or a secondary concussive event occurred during the recovery of the index injury. University of Toronto research ethics board provided ethical approval for this study; participant consent was deemed not required given the low potential for risk/harm and retrospective nature of the study. Although data collection was retrospective with respect to the time of the study, coders were blinded to the main outcome variables when collecting data on exposure variables.

### Aerobic exercise and exposure variables

The primary exposure of interest was the time (days from injury) to the initiation of aerobic exercise following concussion, either self-initiated or physician-prescribed. The time at which aerobic exercise was initiated was captured for all concussion injuries through the use of standardized reporting concussion forms. Self-initiated aerobic exercise was defined as jogging, running, swimming, cycling, or utilization of stationary aerobic equipment.

All physician-prescribed activity followed the graded return to activity guidelines, as per the Berlin Consensus Statement [1]. Physician-prescribed aerobic exercise uniformly commenced with aerobic exercise involving a standardized stationary bike protocol, with minimal head movement/acceleration and increasing levels of exertion and intensity. The standardized stationary bike protocol is as follows: 15 minutes (min) maintaining 100–120 beats per minute (bpm), followed by 30 min at 100–120 bpm, followed by 30 min at 140 bpm, followed by intervals of a 1 min maximal sprint every 5 minutes for a total of 30 min. A minimum of 2 sessions tolerated at each level is recommended prior to progression to the next level. Physician prescribed aerobic exercise was not deferred until symptom resolution, as per the Consensus Statement and growing concussion literature [1].

The time at which the prescribed exercise was *initiated*, as opposed to *prescribed*, was used to define the time to exposure. The time to *first* exposure to aerobic exercise, either self-initiated or physician-prescribed, was used to define the main exposure in individuals who both self-initiated and were prescribed exercise. Day one for the time to initiation of aerobic exercise was defined as within 24 hours of the time of injury and failure to be removed from play was not coded as initiating aerobic exercise.

Data pertaining to additional covariates of interest were collected including age, sex, number of previous concussions, time to first assessment (days), initial symptom severity (based on the symptom checklist from the Sideline Concussion Assessment Tool [SCAT 3 & 5]), loss of consciousness (LOC), post-traumatic amnesia (PTA), history of psychiatric illness, history of headache disorder (including migraines), and history of learning disability (including attention deficit hyperactivity disorder) [18, 19].

### Main outcomes

The main outcomes evaluated for all participants were time (days from injury) to full return to (1) sport and (2) school or work (school/work). This approach allowed for the assessment of recovery within both a predominantly physical (sport) and cognitive (school/work) domain. All acute concussions were serially followed until the individual had fully returned or was cleared to fully return to sport and school/work. The outcomes of interest were defined as the time to (i) full return, as assessed by the sports physician, or (ii) physician clearance for full return. Physician determination of full return to a functional domain is individualized and relies on a combination of symptom state, current and premorbid baseline function, and objective assessments. During data collection, the time to initiation of aerobic exercise was collected blinded to the time to full return to sport and school/work.

### Propensity score stratification

Covariate balancing propensity scores (CBPS) for a continuous treatment (i.e. time-to-aerobic exercise) were estimated to calculate the conditional probability of exposure to the variable of interest accounting for additional observed variables [20]. The potential confounders for propensity score balancing were selected based on literature review and *a piori* hypothesis [21], and included: age, sex, symptom severity, time to first assessment, LOC, PTA, history of psychiatric condition, and history of a headache disorder.

Quintile stratification by propensity score was employed to partially control for systematic differences in the observed variation in the time for individuals to start aerobic exercise post-concussion [22]. It has been estimated that stratifying on the quintiles of propensity score estimates can eliminate approximately 90% of the bias resulting from imbalances in covariate distribution [20, 21]. Quintile strata were included in the final multivariate Cox regression models (see below) as a nominal categorical variable [22]. Within-quintile main effect analyses were not performed due to sample size limitations.

**Fig 1:**
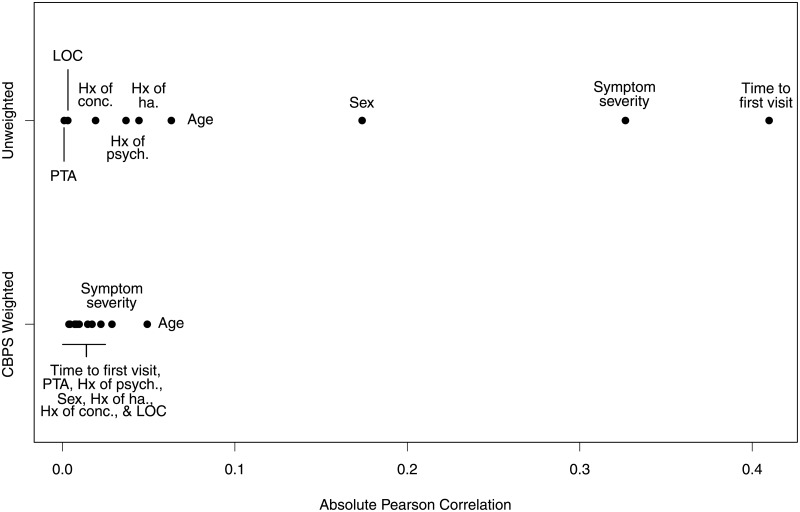
Unweighted and continuous balancing propensity score (CBPS) weighted absolute Pearson correlation between included covariates and time to aerobic exercise.

**Fig 2:**
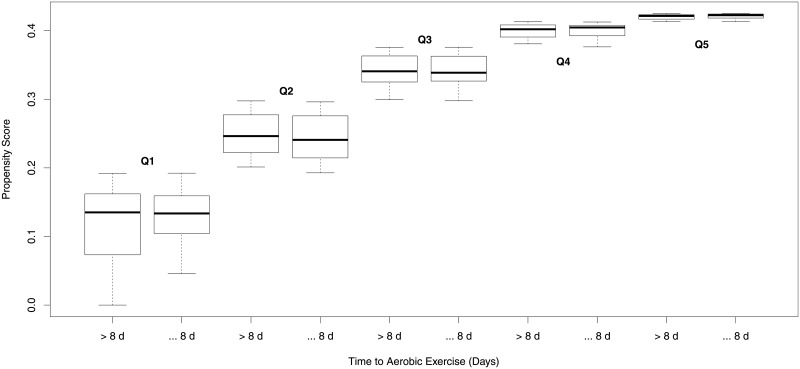
Propensity score values within each propensity score quintile (Q1-Q5) dichotomized by time to aerobic exercise.

Balance diagnostics were carried out to ensure the propensity score model was adequately specified. An unweighted and CBPS-weighted absolute Pearson correlation were calculated for each confounder in the CBPS model (Fig 1). Although the main effects Cox models utilized time to aerobic exercise as a continuous variable, additional balance diagnostics were performed with time to aerobic exercise dichotomized around the median (8 days). These included the estimation of overall and within-quintile standardized mean differences (SMD) and correlation coefficients (Pearson and Point-biserial; S1 Table and Fig 2).

### Data analysis & main effect survival models

Survival analyses were conducted using multivariate Cox proportional hazard models to determine the association between the time-to-aerobic exercise (days) and time (days) to full return to (1) sport and (2) school or work (school/work). The main outcome variables were apportioned to capture information on recovery to a predominantly physical (sport) versus cognitive (school/work) stressor.

Additional covariates of interest were included in the model based on the previous literature and *a priori* hypotheses and included: age, sex, symptom severity, time to first assessment, LOC, PTA, history of psychiatric condition, history of a headache disorder, and a history of a learning disorder. As above, the estimated propensity score strata were included in each model as a nominal categorical variable.

Nonlinearity of the time to aerobic exercise variable was explored using a restricted cubic spline with three knots. A likelihood ratio test demonstrated the full model, with the nonlinear term, differed significantly form the reduced model for both Cox models (p<0.001 and p<0.001, respectively). Moreover, inclusion of this nonlinear term, in addition to stratifying both models by time to first assessment, preserved the proportional hazards assumption for both models (p>0.05). Examination of Martingale and Score residuals did not demonstrate any deviation from linearity or overly influential observations. Variance inflation factors demonstrated no colinearity of any included variables for both models. Predicted hazard-ratios (HR) were calculated to estimate the effect size for the association between predictor variables and the probability of a faster return to sport and school/work.

Zero patients were left censored. Patients who were lost to follow up (n = 23 for full return to sport, n = 15 for full return to school/work) were right censored at the time of the scheduled follow up appointment. All remaining individuals were followed to full recovery.

A significance level of α < 0.05 was used for all tests. The influence of multiplicity of testing was carried out using a false discovery rate (FDR). To note, all tests that achieved an initial significance of α < 0.05 retained significance after FDR adjustment. All analyses were conducted using R software v 3.4.3.

## Results

A total of 253 acute concussions, including 148 males (58.5%), with a median age of 17.0 years (IQR, 15.0–20.0) were included in this study (Table 1). A history of psychiatric illness, headache disorder, and learning disability was identified in 15.4% (n = 39), 9.5% (n = 24), and 9.1% (n = 23) of the study sample, respectively. A total of 126 individuals (49.8%) reported no previous concussion, while the mean number of previous concussions in the sample was 0.8 (SD, 1.0).

**Table 1:** Demographic data for included study participants.

Variables	Number (%) or median (IQR)	
Age (years)	17.0	(15.0–20.0)
Symptom severity	26.0	(12.0–44.0)
Sex		
Male	148	(58.5)
Female	105	(41.5)
Sport		
Hockey	63	(24.9)
Rugby	31	(12.3)
Football	29	(11.5)
Soccer	22	(8.7)
Basketball	18	(7.1)
Skiing/snowboarding	15	(5.9)
Volleyball	14	(5.5)
Other	61	(24.1)
History of psychiatric disorder	39	(15.4)
History of headache disorder	24	(9.5)
History of learning disability	23	(9.1)
Loss of consciousness	12	(4.7)
Post-traumatic amnesia	21	(8.3)
Number of previous concussions		
0	126	(49.8)
1	74	(29.2)
2	35	(13.8)
≥3	18	(7.1)
mean (SD)	0.8	(1.0)
Time to first assessment (days)		
≤2	59	(23.3)
3 to 4	87	(34.4)
5 to 6	36	(14.2)
7 to 8	27	(10.7)
9 to 10	17	(6.7)
>10	27	(10.7)
median (IQR)	4.0	(3.0–7.0)
Time to aerobic exercise (days)		
≤2	8	(3.2)
3 to 4	52	(20.6)
5 to 6	35	(13.8)
7 to 8	33	(13.0)
9 to 10	32	(12.6)
>10	93	(36.8)
median (IQR)	8.0	(5.0–12.0)
Time to full sport (days)	28.0	(17.0–43.0)
Time to full school/work (days)	23.0	(15.0–36.0)

IQR, Inter-quartile range; SD, standard deviation

The majority of the injuries occurred during ice hockey (n = 63, 24.9%), followed by rugby (n = 31, 12.3%), football (n = 29, 11.5%), and soccer (n = 22, 8.7%). Loss of consciousness and PTA were reported in 4.7% and 8.3% of the injuries, respectively. The median time to first assessment was 4.0 days (IQR, 3.0–7.0) and the median symptom severity score was 26.0 (IQR, 12.0–44.0). The median time to aerobic exercise was 8.0 days (IQR, 5.0–12.0) post-injury. The median time to full return to sport and school/work was 28.0 days (IQR, 17.0–43.0) and 23.0 days (IQR, 15.0–36.0), respectively.

### Main effect survival analyses

For each successive day in delay to initiation of aerobic exercise, individuals had a less favourable recovery trajectory. Moreover, given the nonlinear association between time to aerobic exercise and concussion recovery, a stronger protective effect for earlier aerobic exercise was observed.

**Fig 3:**
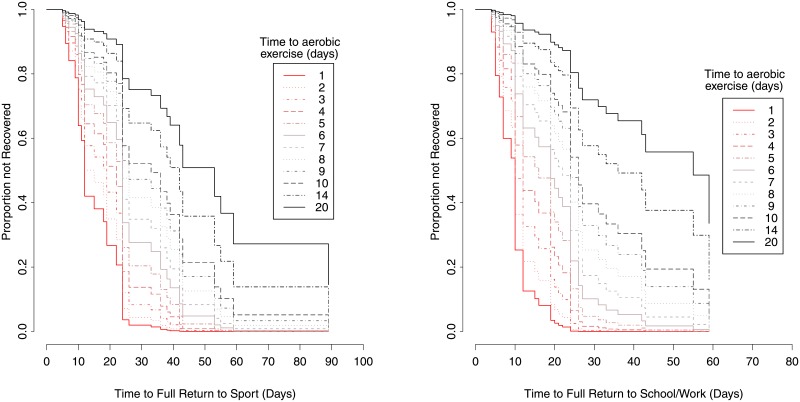
Kaplan-Meier survival curves for time to full return to A. sport and B. school/work based on the time to initiation of aerobic exercise, after adjustment for all covariates including propensity score strata.

For example, initiating aerobic exercise on 3, 5, 7, and 14 days post-injury was associated with a respective 36.5% (HR, 0.63; 95% CI, 0.53–0.76), 59.5% (HR, 0.41; 95% CI, 0.28–0.58), 73.2% (HR, 0.27; 95% CI, 0.16–0.45), and 88.9% (HR, 0.11; 95% CI, 0.06–0.22) reduced probability of faster full return to sport compared to initiating aerobic exercise within 1 day post injury (p<0.001; Table 2), after adjusting for all other covariates including quintiled propensity score strata (Fig 3A).

**Table 2:** Multivariate cox regression models for the effect of the included exposure variables on predicted time to full return to sport and school/work.

Included variables	Time to full return to sport			Time to full return to school/work		
	HR	95% CI	p value	HR	95% CI	p value
Time to aerobic exercise (days)						
1	-	-	<0.001^a^	-	-	<0.001^b^
2	0.80	(0.73, 0.87)		0.74	(0.67, 0.81)	
3	0.63	(0.53, 0.76)		0.54	(0.44, 0.66)	
4	0.51	(0.38, 0.67)		0.40	(0.29, 0.54)	
5	0.41	(0.28, 0.58)		0.29	(0.20, 0.44)	
6	0.33	(0.21, 0.51)		0.22	(0.14, 0.36)	
7	0.27	(0.16, 0.45)		0.17	(0.10, 0.30)	
8	0.22	(0.13, 0.40)		0.13	(0.07, 0.25)	
9	0.19	(0.10, 0.35)		0.11	(0.05, 0.21)	
10	0.17	(0.09, 0.32)		0.09	(0.04, 0.18)	
14	0.11	(0.06, 0.22)		0.05	(0.03, 0.11)	
Age (+5 years)	1.01	(0.83, 1.23)	0.927	0.88	(0.73, 1.06)	0.193
Sex (female vs male)	0.83	(0.59, 1.17)	0.283	1.20	(0.68, 2.11)	0.639
Symptom severity (+ 4 points) ^c^	0.92	(0.81, 1.03)	0.158	0.86	(0.77, 0.97)	0.015
Concussion history (no.)	0.84	(0.71, 0.99)	0.041	0.84	(0.71, 0.99)	0.038
LOC	0.30	(0.13, 0.66)	0.003	0.78	(0.38, 1.59)	0.492
PTA	1.10	(0.62, 1.95)	0.740	1.02	(0.57, 1.82)	0.958
Psychiatric condition	0.75	(0.48, 1.18)	0.213	0.80	(0.51, 1.26)	0.331
Headache condition	0.76	(0.44, 1.29)	0.309	0.78	(0.46, 1.34)	0.369
Learning disability	1.19	(0.67, 2.12)	0.548	1.09	(0.76, 1.56)	0.532
Propensity score strata (Q1: Q5)^d^			>0.05			>0.05

CI, confidence interval; HR, hazard ratio; LOC, loss of consciousness; No., number; PTA, post-traumatic amnesia; Q, quintile; vs, versus

^a^ 0.021 for nonlinear function;

^b^ 0.002 for nonlinear function

^c^ Score out of 132 based on symptom checklist from SCAT3&5

^d^ Nominal variable with 5 levels

Moreover, initiating aerobic exercise on 3, 5, 7, and 14 days post-injury was associated with a respective 45.9% (HR, 0.54; 95% CI, 0.44–0.66), 70.5% (HR, 0.29; 95% CI 0.20–0.44), 83.1% (HR, 0.17; 95% CI, 0.10–0.30), and 94.7% (HR, 0.05; 95% CI, 0.03–0.11) reduced probability of faster full return to school/work compared to initiating aerobic exercise within 1 day post injury (p<0.001; Table 2), after adjusting for all other covariates including quintiled propensity score strata (Fig 3B).

The number of previous concussions and LOC had a deleterious effect on time to return to sport (HR, 0.84; 95% CI, 0.71–0.99; p = 0.041 and HR, 0.30; 95% CI, 0.13–0.66; p = 0.003; respectively), after adjusting for all other covariates. The number of previous concussions and symptom severity had a deleterious effect on time to return to school/work (HR, 0.84; 95% CI, 0.71–0.99; p = 0.038 and HR, 0.86; 95% CI, 0.77–0.97; p = 0.015; respectively). Age, sex, PTA, propensity score strata, history of psychiatric condition, headache condition, and learning disability were not associated with either return to sport or school/work.

## Discussion

This study examined the relationship between the time to initiate aerobic exercise following an acute concussion and the time to full return to sport and school/work with consideration of known risk modifiers for concussion. Shorter time to initiation of aerobic exercise post-concussion was associated with faster full return to sport and school/work. Furthermore, all individuals were symptomatic at the time of initiating aerobic exercise; supporting the current agreement that aerobic exercise is safe and potentially protective in symptomatic individuals.

Our findings provide evidence for the paradigm shift towards an active rehabilitative approach to the management of acute concussion. Historically, the standard of care after concussion was largely predicated upon experimental animal studies, which identified a decrease in energy availability in the brain resulting from neurometabolic disruption [23]. From this, it was speculated that activity after an injury might exacerbate this metabolic dysfunction, increase symptoms, and delay recovery [4, 24]; however, empirical evidence in human subjects has not supported this position. Complete or prolonged rest following concussion has been shown to increase symptom burden and delay recovery [3–5, 7, 9] and growing evidence is supporting the introduction of earlier activity in the management of concussion [11]. Despite the reported benefits of aerobic exercise for those with persistent symptoms [8, 10–14], a recent systematic review and meta-analysis only identified two studies examining the benefit of physical exercise following acute concussion (<5 days) [12]. Nonetheless, Lal and colleagues found “early physical activity” was associated with a lower risk of persistent post-concussion symptoms [12]. Moreover, moderate levels of exertion (slow jogging and sports practice) during concussion recovery demonstrated better neurocognitive outcomes compared to low or high intensity activities [8].

Graded exercise programs have demonstrated positive impacts on many other central nervous system and medical conditions such as stroke [25], depression [26], anxiety [27], fibromyalgia [28], chronic fatigue syndrome [29], and cognitive impairment [4, 30, 31]. Moreover, aerobic exercise has a protective physiologic effect on multiple organ systems, including the cardiovascular and pulmonary-, central nervous-, and neuroendocrine- systems and is hypothesized to effectuate its positive role in brain health through the promotion of neurogeneisis, angiogenesis, and neurotrophic growth factors [32].

Despite our present study findings, which contribute to the growing understanding of the benefits of activity following acute concussion, more evidence is required to provide a detailed evidence-based exercise prescription. All exercise prescriptions require several elements outlined by the ‘FITT’ principle, including the frequency, intensity, time, and type of exercise [33]. The FITT prescription represents an important construct for exercise prescription; however, is limited for prescribing exercise post-injury, as it does not elaborate on when an individual should initiate exercise post injury. Exercise prescription following any injury (including concussion) should additionally detail recommendations pertaining to the timing of initiation of exercise post-injury. Therefore, we propose a “FITTT” exercise prescription be employed for concussion, which includes the aforementioned components with an additional description of the “time post-injury” recommendation.

This study is consistent with the current understanding of the role of aerobic exercise following concussion and provides additional support for earlier (within 1 week) introduction of aerobic exercise following acute concussion. This study did not allow for comprehensive assessment of exercise intensity, type, and symptom response; however, all recommendations for prescribed exercise involved low impact (stationary bike) sub-symptom threshold exercise. Further studies, in the form of randomized controlled trials, are required to elucidate more detailed recommendations for exercise prescriptions post-concussion.

Additional risk modifiers for concussion recovery were explored through this study [18, 19]. A history of concussion, higher symptom burden, and loss of consciousness were associated with a prolonged recovery, which is consistent with the current understanding surrounding these risk modifiers [18, 19]. Increasing awareness and attention is being placed on sex differences and outcomes following concussion [18, 19]. Female athletes have been demonstrated to have an increased risk of sustaining a concussion and report higher symptom burden compared to male counterparts [18, 19, 34]; however, the evidence on sex-specific recovery measures is mixed [18]. The results of the current study did not observe a differential recovery pattern based on sex. Furthermore, age, learning disability, psychiatric history, headache disorder, and post-traumatic amnesia did not predict recovery from acute concussion based on the results of this study.

This study is limited by several factors. Physician-recommended aerobic exercise was consistent during the study time period and involved a stationary bike protocol with graded increases in intensity and duration. However, greater variability in the type of exercise was observed in individuals who self-initiated aerobic exercise post-concussion, with further difficulty in defining the exercise intensity. Moreover, the duration and intensity of self-imitated exercise were not captured in this study. Although efforts were made to reduce the bias in the distribution of variables associated with the time at which individuals initiated aerobic exercise, it is recognized residual bias may still exist even after stratification by propensity score [20, 22]. Statistical modelling and predicted effect sizes of the benefits of initiating aerobic exercise were demonstrated in this study; however, further examination into the initiation of aerobic exercise within 28–48 hours of concussion should be undertaken to substantiate these observations.

## Conclusion

This study investigated the association between the time to initiate aerobic exercise following acute symptomatic concussion and the time to full return to sport and school or work with consideration of known risk modifiers for concussion. Early aerobic exercise was shown to be safe and protective in improving recovery time following acute concussion.

## Supporting information

S1 Table Balance diagnostics for the distribution of confounders on dichotomized time to aerobic exercise based on propensity score quintile stratum. Cor, correlation; IQR, inter-quartile range; Q, propensity score quintile stratum; SMD, standardized mean difference; sd, standard deviation. ^a^, Pearson for continuous variables & Point-biserial for binary.
(DOCX)
