# Supplementary material for: Earlier time to aerobic exercise is associated with faster recovery following acute sport concussion
Source: PLoS One. 2018 Apr 18;13(4):e0196062. doi: 10.1371/journal.pone.0196062 (PMC5905975; doi:10.1371/journal.pone.0196062)
Supplement: S1 Table — Cor, correlation; IQR, inter-quartile range; Q, propensity score quintile stratum; SMD, standardized mean difference; sd, standard deviation. a, Pearson for continuous variables & Point-biserial for binary. (DOCX) [file pone.0196062.s001.docx]

| **Confounders** | **Unbalanced [No. (%) or median (IQR)]** | | | | | |  | **Stratified by Propensity Score [No. (%) or median (IQR)]** | | | | | | | | | | | | |
| --- | --- | --- | --- | --- | --- | --- | --- | --- | --- | --- | --- | --- | --- | --- | --- | --- | --- | --- | --- | --- |
|  |  |  |  |  |  |  |  | **Q1 (n=51)** | | | | | |  | **Q2 (n=50)** | | | | | |
|  | **Time to Aerobic Exercise** | | | | | **Cor. ^a^** |  | **Time to Aerobic Exercise** | | | | | **Cor. ^a^** |  | **Time to Aerobic Exercise** | | | | | **Cor. ^a^** |
|  | **≤8 days (n =128)** | | **>8 days (n = 125)** | | **SMD** |  |  | **≤8 days (n =18)** | | **>8 days (n = 33)** | | **SMD** |  |  | **≤8 days (n =24)** | | **>8 days (n =26)** | | **SMD** |  |
| **Time to full sport (days)** | **19.0** | **(12.0-28.0)** | **39.0** | **(28.0-52.0)** | -0.85 | 0.45 |  | 24.5 | (14.3-39.0) | 44.0 | (25.0-63.0) | -0.83 | 0.54 |  | 21.0 | (11.0-28.8) | 41.0 | (34.0-51.3) | -1.22 | 0.42 |
| **Time to full school/work (days)** | **16.0** | **(10.0-24.0)** | **33.0** | **(24.4-44.4)** | -1.12 | 0.58 |  | 22.0 | (12.5-28.5) | 36.0 | (24.0-51.0) | -1.14 | 0.72 |  | 17.5 | (10.0-24.0) | 34.5 | (28.3-44.0) | -1.31 | 0.43 |
|  |  |  |  |  |  |  |  |  |  |  |  |  |  |  |  |  |  |  |  |  |
| Age (years) | 17.0 | (14.0-21.0) | 17.0 | (15.0-20.0) | 0.12 | -0.06 |  | 20.0 | (16.0-21.8) | 17.0 | (16.0-20.0) | 0.48 | -0.08 |  | 18.0 | (15.8-21.3) | 18.5 | (16.0-20.0) | 0.00 | -0.14 |
| Symptom severity | 15.5 | (7.0-30.3) | 32.0 | (21.0-49.0) | -0.73 | 0.33 |  | 28.5 | (11.5-48.8) | 31.0 | (16.0-53.0) | -0.21 | 0.09 |  | 28.0 | (11.0-54.3) | 34.0 | (22.3-61.3) | -0.31 | 0.23 |
| Time to first visit (days) | 3.0 | (2.0-4.0) | 6.0 | (3.0-9.0) | -0.91 | 0.41 |  | 3.5 | (2.0-5.5) | 5.0 | (3.0-11.0) | -0.58 | 0.02 |  | 3.5 | (2.0-4.0) | 7.5 | (3.5-10.8) | -1.24 | 0.53 |
| Number of previous concussion (mean, sd) | 0.8 | (1.04) | 0.8 | (0.98) | 0.01 | 0.02 |  | 1.2 | (3.70) | 0.9 | (5.13) | 0.25 | -0.05 |  | 0.9 | (1.59) | 0.9 | (4.26) | -0.05 | 0.03 |
| Sex (male) | 88.0 | (68.8) | 60.0 | (48.0) | 0.43 | 0.17 |  | 7.0 | (38.9) | 14.0 | (42.4) | -0.07 | -0.04 |  | 19.0 | (79.2) | 16.0 | (61.5) | 0.39 | 0.15 |
| History of psychiatric disorder | 16.0 | (12.5) | 23.0 | (18.4) | -0.16 | -0.04 |  | 6.0 | (33.3) | 6.0 | (18.2) | 0.35 | 0.21 |  | 2.0 | (8.3) | 4.0 | (15.4) | -0.22 | -0.10 |
| History of headache disorder | 9.0 | (7.0) | 15.0 | (12.0) | -0.17 | 0.04 |  | 2.0 | (11.1) | 1.0 | (3.0) | 0.32 | -0.12 |  | 2.0 | (8.3) | 5.0 | (19.2) | -0.32 | 0.07 |
| History of learning disorder | 10.0 | (7.8) | 13.0 | (10.4) | -0.09 | -0.08 |  | 3.0 | (16.7) | 5.0 | (15.2) | 0.04 | -0.16 |  | 1.0 | (4.2) | 3.0 | (11.5) | -0.28 | -0.14 |
| Loss of consciousness | 6.0 | (4.7) | 6.0 | (4.8) | -0.01 | 0.00 |  | 0.0 | (0.0) | 2.0 | (6.1) | -0.36 | -0.02 |  | 1.0 | (4.2) | 2.0 | (7.7) | -0.15 | -0.26 |
| Post-traumatic amnesia | 9.0 | (7.0) | 12.0 | (9.6) | -0.09 | 0.00 |  | 1.0 | (5.6) | 2.0 | (6.1) | -0.02 | 0.04 |  | 3.0 | (12.5) | 1.0 | (3.8) | 0.32 | 0.18 |

**Supplemental Table. Balance diagnostics for the distribution of confounders on dichotomized time-to-aerobic exercise based on propensity score quintile stratum.**

Cor, correlation; IQR, inter-quartile range; Q, propensity score quintile stratum; SMD, standardized mean difference; sd, standard deviation

^a^, Pearson for continuous variables & Point-biserial for binary

**Supplemental Table. Balance diagnostics for the distribution of confounders on dichotomized time-to-aerobic exercise based on propensity score quintile stratum.**

|  | | | | | | | | | | | | | | | | | | | |  |
| --- | --- | --- | --- | --- | --- | --- | --- | --- | --- | --- | --- | --- | --- | --- | --- | --- | --- | --- | --- | --- |
| **Confounders** | **Stratified by Propensity Score [No. (%) or median (IQR)]** | | | | | | | | | | | | | | | | | | | |
|  | **Q3 (n=51)** | | | | | |  | **Q4 (n=50)** | | | | | |  | **Q5 (n=51)** | | | | | |
|  | **Time to Aerobic Exercise** | | | | | **Cor. ^a^** |  | **Time to Aerobic Exercise** | | | | | **Cor. ^a^** |  | **Time to Aerobic Exercise** | | | | | **Cor. ^a^** |
|  | **≤8 days (n = 25)** | | **>8 days (n =26)** | | **SMD** |  |  | **≤8 days (n = 31)** | | **>8 days (n =19)** | | **SMD** |  |  | **≤8 days (n = 30)** | | **>8 days (n =21)** | | **SMD** |  |
| **Time to full sport (days)** | 16.0 | (11.0-29.0) | 38.5 | (22.3-47.0) | -0.92 | 0.41 |  | 17.0 | (11.0-24.5) | 38.0 | (29.5-49.5) | -0.70 | 0.38 |  | 18.5 | (12.5-26.3) | 35.0 | (28.0-47.0) | -0.93 | 0.49 |
| **Time to full school/work (days)** | 15.0 | (8.0-22.0) | 32.0 | (21.3-42.0) | -1.44 | 0.70 |  | 12.0 | (9.0-20.5) | 30.0 | (21.0-41.5) | -0.89 | 0.55 |  | 16.0 | (12.0-21.8) | 33.0 | (20.0-41.0) | -1.07 | 0.59 |
|  |  |  |  |  |  |  |  |  |  |  |  |  |  |  |  |  |  |  |  |  |
| Age (years) | 15.0 | (14.0-19.0) | 15.0 | (14.0-18.0) | 0.17 | -0.06 |  | 17.0 | (14.0-18.5) | 17.0 | (15.0-19.0) | -0.07 | 0.04 |  | 17.0 | (15.0-21.0) | 17.0 | (16.0-20.0) | 0.11 | -0.23 |
| Symptom severity | 17.0 | (11.0-26.0) | 31.5 | (19.5-44.0) | -0.79 | 0.46 |  | 10.0 | (2.0-18.0) | 32.0 | (26.5-45.0) | -1.29 | 0.53 |  | 13.0 | (7.3-20.0) | 33.0 | (7.3-(20.0) | -1.39 | 0.44 |
| Time to first visit (days) | 3.0 | (2.0-4.0) | 7.0 | (3.0-8.0) | -0.86 | 0.61 |  | 4.0 | (2.0-4.0) | 6.0 | (4.0-9.0) | -1.15 | 0.64 |  | 3.0 | (3.0-5.8) | 5.0 | (5.0-7.0) | -0.89 | 0.680 |
| Number of previous concussion (mean, sd) | 0.7 | (1.73) | 0.6 | (3.47) | 0.15 | -0.15 |  | 0.5 | (1.63) | 0.8 | (3.54) | -0.33 | 0.21 |  | 0.9 | (1.90) | 0.8 | (3.46) | 0.12 | -0.01 |
| Sex (male) | 23.0 | (92.0) | 9.0 | (34.6) | 1.48 | 0.55 |  | 18.0 | (58.1) | 12.0 | (63.2) | -0.10 | 0.09 |  | 21.0 | (70.0) | 9.0 | (42.9) | 0.57 | 0.15 |
| History of psychiatric disorder | 2.0 | (8.0) | 5.0 | (19.2) | -0.33 | -0.20 |  | 2.0 | (6.5) | 4.0 | (21.1) | -0.43 | -0.22 |  | 4.0 | (13.3) | 4.0 | (19.0) | -0.16 | 0.01 |
| History of headache disorder | 1.0 | (4.0) | 4.0 | (15.4) | -0.39 | 0.14 |  | 1.0 | (3.2) | 2.0 | (10.5) | -0.29 | 0.21 |  | 3.0 | (10.0) | 3.0 | (14.3) | -0.13 | 0.05 |
| History of learning disorder | 2.0 | (8.0) | 4.0 | (15.4) | -0.23 | -0.01 |  | 0.0 | (0.0) | 0.0 | (0.0) |  |  |  | 4.0 | (13.3) | 1.0 | (4.8) | 0.302 | 0.21 |
| Loss of consciousness | 2.0 | (8.0) | 1.0 | (3.9) | 0.18 | 0.11 |  | 3.0 | (9.7) | 0.0 | (0.0) | 0.46 | 0.25 |  | 0.0 | (0.0) | 1.0 | (4.8) | -0.32 | -0.02 |
| Post-traumatic amnesia | 2.0 | (8.0) | 2.0 | (7.7) | 0.01 | -0.15 |  | 1.0 | (3.2) | 3.0 | (15.8) | -0.44 | -0.20 |  | 2.0 | (6.7) | 4.0 | (19.0) | -0.38 | 0.00 |
|  | | | | | | | | | | | | | | | | | | | |  |
|  | | | | | | | | | | | | | | | | | | | |  |

Cor, correlation; IQR, inter-quartile range; Q, propensity score quintile stratum; SMD, standardized mean difference; sd, standard deviation

^a^, Pearson for continuous variables & Point-biserial for binary
